# Pregnancy outcomes after ultrasound- and physical examination-indicated cervical cerclage: a retrospective cohort study

**DOI:** 10.1007/s00404-026-08437-9

**Published:** 2026-04-21

**Authors:** Chen Manor-Bar, Ettie Piura, Zvi Klein, David Rubinshtein, Tal Biron-Shental, Hanoch Schreiber, Michal Kovo, Yair Daykan, Nissim Arbib

**Affiliations:** 1 Department of Obstetrics and Gynecology, Meir Medical Center, 59 Tchernichovsky St., 4428164 Kfar Saba, Israel; 2 Gray Faculty of Medical and Health Sciences, Tel Aviv University, Tel Aviv, Israel; 3 Faculty of Health Sciences, Ben-Gurion University of the Negev, Beer-Sheva, Israel; 4 Obstetrics and Gynecology Department, “Shamir” (“Assaf Harofeh”) Medical Center, Tzrifin, Israel

**Keywords:** Emergency cervical cerclage, Cervical insufficiency, Ultrasound-indicated cerclage, Physical examination-indicated cerclage, Preterm birth

## Abstract

**Objective:**

To compare pregnancy outcomes after emergency cervical cerclage according to indication: ultrasound-indicated versus physical examination-indicated cerclage.

**Methods:**

This retrospective cohort study included singleton pregnancies that underwent emergency cerclage using the McDonald technique at 18–23.6 weeks of gestation at a single tertiary center between 2017 and 2023. Cases were classified according to indication: ultrasound-indicated cerclage (cervical length ≤ 25 mm with a closed cervix) or physical examination-indicated cerclage (painless cervical dilation ≥ 1 cm with visible membranes). The primary outcome was early preterm birth, defined as delivery before 34 weeks of gestation. Secondary outcomes included pregnancy prolongation following cerclage and other obstetric outcomes.

**Results:**

Among 52 emergency cerclage cases, 39 patients underwent ultrasound-indicated cerclage and 13 underwent physical examination-indicated cerclage. Maternal characteristics and gestational age at the time of the procedure were similar between groups. Previous hysteroscopy was more common in the physical examination-indicated cerclage group (*p* = 0.035). Overall, 84.6% of patients (44/52) delivered after 34 weeks of gestation, with no significant difference in gestational age at delivery between groups (*p* = 0.886). Pregnancy prolongation did not differ significantly between groups (15.5 ± 4.2 vs. 13.3 ± 6.2 weeks; *p* = 0.168). In multivariable analysis, cerclage indication was not independently associated with early preterm birth, whereas pre-cerclage cervical length remained an independent predictor (aOR 0.73, *p* = 0.02).

**Conclusion:**

Emergency cervical cerclage was associated with substantial pregnancy prolongation. No statistically significant differences were observed between indications, and pre-cerclage cervical length was the only independent predictor of early preterm birth. These findings support, but do not confirm, a potential pathophysiologic continuum of cervical insufficiency.

## Whatn does this study add to the clinical work?

**Table:** 

**Condensation**
Emergency cervical cerclage was associated with substantial pregnancy prolongation. Pregnancy outcomes showed no statistically significant differences between ultrasound- and physical examination-indicated cerclage.
**Why was this study conducted?**
To compare pregnancy outcomes after emergency cervical cerclage according to indication, ultrasound-detected cervical shortening versus cervical dilation on physical examination.
**What are the key findings?**
Pregnancy outcomes showed no statistically significant differences between ultrasound and physical examination-indicated cerclage. Pre-cerclage cervical length, rather than indication category, was the only independent predictor of early preterm birth.
**What does this study add to what is already known?**
These findings support the concept that ultrasound-detected cervical shortening and cervical dilation may represent different stages of the same pathophysiologic continuum of cervical insufficiency.

## Introduction

Cervical insufficiency (CI) is defined by painless cervical dilation, resulting in second-trimester pregnancy loss or preterm birth (PTB), and is attributed to structural cervical weakness [1]. It affects approximately 1% of all pregnancies and up to 8% of women with recurrent mid-trimester pregnancy losses, and contributes significantly to PTB, a leading cause of neonatal morbidity and mortality worldwide, affecting approximately 10% of pregnancies [1, 2]. Importantly, CI represents a spectrum of disease severity, ranging from asymptomatic cervical shortening to advanced cervical dilation [1]. Primary interventions to reduce the risk of second-trimester fetal loss and early PTB include vaginal progesterone and cervical cerclage [3–6].

According to the professional guidelines [7–9], cervical cerclage is considered in asymptomatic women with a singleton pregnancy who have a history of spontaneous PTB and a short cervix (< 25 mm) identified on mid-trimester ultrasound. The procedure is intended to provide mechanical support to the cervix and reduce the risk of recurrent PTB. However, in cases without a prior PTB, vaginal progesterone is usually recommended when a short cervix is detected [7–12].

The terminology regarding cerclage indications varies in the literature; “Rescue cervical cerclage” has been inconsistently used for cases with cervical dilation and visible or protruding membranes, whereas “Emergency cervical cerclage (ECC)” is often applied more broadly, encompassing both physical examination-indicated and ultrasound-indicated cerclage [13–15]. In clinical practice, these categories may overlap, as cervical shortening identified on ultrasound can progress to dilation confirmed on physical examination.

Reported success rates for ECC range from 60 to 80%, with a mean pregnancy prolongation of approximately 4–7 weeks, although outcomes vary substantially according to the clinical scenario and patient characteristics [16–19].

Cervical shortening detected on mid-trimester ultrasound may represent an early stage of the same pathological process that later manifests as painless cervical dilation on physical examination. Studies have shown that progressively shorter cervical length is associated with subsequent asymptomatic cervical dilation and an increased risk of preterm birth, with the risk increasing as cervical length decreases [20–22]. These observations support the concept that cervical shortening and cervical dilation may represent sequential manifestations of the same underlying process of cervical insufficiency. In this study, we compared pregnancy outcomes after ultrasound-indicated and physical examination-indicated emergency cerclage, hypothesizing that cervical shortening detected on ultrasound and cervical dilation on physical examination may represent different stages of the same pathophysiologic continuum of cervical insufficiency**.**

## Materials and methods

This retrospective study analyzed data from all women with singleton pregnancies, with and without a history of preterm birth, who underwent ECC between 18 and 23 + 6 weeks of gestation from 2017 to 2023 at a single tertiary medical center. All data were collected from electronic medical records.

Women were included if they were referred due to painless cervical dilation or mid-trimester ultrasound findings of cervical shortening, regardless of prior progesterone treatment. Physical examination-indicated cerclage was defined as cervical dilation ≥ 1 cm on manual exam and ultrasound-indicated cerclage was defined as a cervical length ≤ 25 mm and a closed cervix by physical examination, as defined by professional society guidelines [23]. In accordance with the Israeli Society of Obstetrics and Gynecology guidelines, cervical length was measured during the second trimester as part of routine monitoring in low-risk pregnancies, with a transvaginal cervical length under 25 mm before 24 weeks considered shortened. Patients with a twin pregnancy, clinical signs of chorioamnionitis, lower abdominal pain, or vaginal bleeding were excluded. Amniocentesis was not routinely offered. Cerclage was performed in cases of a short cervix with a prior preterm birth or late abortion suggestive of cervical insufficiency. In nulliparous women, cases were included if cervical length was < 15 mm during the second trimester or if cervical dilation ≥ 1 cm was detected in the absence of symptoms.

### Cervical cerclage

As part of our departmental protocol before placing a cerclage, all participants underwent a comprehensive evaluation, including physical examination, transvaginal ultrasound for cervical length assessment (conducted by skilled obstetricians or certified technicians), tocography to rule out uterine activity, and complete blood count and CRP, to screen for infection. The McDonald cerclage technique, using a 5-mm Mersilene® polyester fiber suture, was uniformly performed by one of three experienced clinicians. Our method involves placing sutures at four points on the cervix, with the knot tied at the 1 o’clock position. In cases where membrane exposure was identified on physical examination, patients were positioned in the Trendelenburg position, and the exposed membranes were reduced using a Foley catheter to apply gentle pressure. Following the procedure, all patients were monitored in the hospital for 24 h. During hospitalization, patients received 1–2 g of prophylactic cefazolin and 50 mg of oral indomethacin. Upon discharge, all patients were prescribed daily vaginal progesterone 200 mg (utrogestan) until 37 weeks of gestation; however, documented use varied among patients.

Patients were divided into two comparison groups based on the indication for cerclage placement: ultrasound-indicated versus physical examination-indicated cerclage.

### Outcome

#### Primary outcome

Early PTB (< 34 weeks).

#### Secondary outcomes

Pregnancy prolongation, gestational age at delivery, and obstetric complications**.**

### Missing data

Missing data were minimal for any variable, less than 5%. Complete case analysis was performed, with participants excluded from specific analyses if data for relevant variables were unavailable.

### Ethics

The study was approved by the local Institutional Review Board (IRB #0088/16). Patient consent was waived due to the retrospective nature of the study.

### Statistical analysis

Nominal data were reported as frequencies and percentages. Continuous variables were presented as mean ± standard deviation (SD). Categorical data were analyzed using the chi-square or Fisher’s exact test, while continuous variables were compared using the t-test or the Mann–Whitney *U* test, depending on data distribution. A *p*-value of < 0.05 was considered statistically significant. All analyses were conducted using SPSS software, version 26 (IBM Corp, Armonk, NY).

Multivariable logistic regression analysis was performed to identify independent predictors of early preterm birth. Variables were selected based on clinical relevance and included maternal age, parity, gestational age at cerclage placement, prior preterm birth, and history of hysteroscopy.

No formal a priori power calculation was performed, as this retrospective study included all eligible cases during the study period.

## Results

During the study period, 57 patients were assessed for eligibility, of whom 5 were excluded (including twin pregnancies, suspected infection, and cervical dilation > 4 cm), leaving 52 patients for final analysis. Among them, 39 (75%) underwent ultrasound-indicated cerclage and 13 (25%) physical examination-indicated cerclage.

Demographic characteristics are presented in Table 1 and were similar between the two groups. There were no significant differences in maternal age, gravidity, parity, smoking status, history of previous abortion, previous PTB, chronic disease, method of conception, BMI (kg/m^2^), loop electrosurgical excision procedure, or mode of delivery between the two groups. Notably, the overall rate of cesarean delivery in the study population was 36.5% (19/52), higher than the overall average in our hospital (21%). A higher percentage of patients in the physical-exam group had a history of at least one hysteroscopy compared to the ultrasound-indicated cerclage group (46.2%, 6/13 vs. 25.6%, 10/39; *p* = 0.035).

**Table 1:** Patient characteristics stratified by cervical cerclage indication

Characteristic	Cerclage indication		*P*-value
	Ultrasound (*n* = 39)	Physical examination (*n* = 13)	
Age (years)	32.7 ± 8.2	33.5 ± 6.1	0.766
Gravidity (n)	3.28 ± 2.3	2.9 ± 1.5	0.606
Parity (n)	1.03 ± 1.2	0.77 ± 1.0	0.506
Body mass index (kg/m^2^)	26.4 ± 4.1	27.8 ± 7.6	0.419
Mode of conception			
Spontaneous	29 (78.4%)	8 (66.7%)	0.377
In vitro fertilization	10 (21.6%)	5 (33.3%)	
Smoking	2 (5.1%)	0	1
Previous abortion	1.2 ± 1.8	1.23 ± 1.4	0.962
Prior PTB < 34 weeks	19 (49%)	3 (23%)	0.299
Chronic disease*	4 (10.2%)	1 (7.7%)	1.000
Loop electrosurgical excision procedure	2 (5.1%)	2 (15.3%)	0.493
Cervical dilation procedures			
Dilation and curettage	0.82 ± 1.25	1 ± 1.04	0.206
Hysteroscopy	10 (25.6%)	6 (46.2%)	0.035
Mode of delivery			
Normal vaginal delivery	17 (43.6%)	7 (53.8%)	0.943
Cesarean section	16 (41%)	3 (23%)	
Vacuum extraction	6 (15.3%)	3 (23%)	

*PTB* preterm birth

Categorical variables are presented as n (%), and continuous variables as mean ± standard deviation (SD). Mode of delivery refers to the current pregnancy. Chronic diseases include gestational diabetes mellitus, diabetes mellitus, hypertensive disorders, asthma, and thyroid disease

Characteristics related to the cerclage are presented in Table 2. There were no differences between groups in gestational age at cerclage placement, timing of suture removal, gestational age at delivery, cervical length post-cerclage, cerclage-to-delivery interval, or the rate of patients treated with vaginal progesterone before and after cerclage. The ultrasound group had a longer cervical length at diagnosis than the physical examination group (13.7 ± 7.3 mm vs. 6.2 ± 5.0 mm; *p* = 0.008). There were no procedure-related complications, such as preterm premature rupture of membranes, chorioamnionitis, increased vaginal bleeding, or cervical tear, in either group.

**Table 2:** Cerclage-related characteristics

Characteristic	Cerclage indication		*P* value (< 0.05)
	Ultrasound (*n* = 39)	Physical examination (*n* = 13)	
Gestational age at cerclage (wk)	20.2 ± 1.93	21.2 ± 1.72	0.131
Suture removal (wk)	35.3 ± 3.6	33.9 ± 4.4	0.272
Gestational age at delivery (wk)	36.3 ± 4.1	36.1 ± 5.5	0.886
Cervical length before (mm)	13.7 ± 7.3	6.2 ± 5.0	0.008
Cervical length after (mm)	24.6 ± 8.8	22.4 ± 8.2	0.421
Delta cervical length (mm)	10.9 ± 9.4	16.2 ± 8.0	0.076
Cerclage-to-delivery interval (wk)	15.5 ± 4.2	13.3 ± 6.2	0.168
Progesterone before cerclage	33 (84.6%)	13 (100%)	0.752
Progesterone after cerclage	26 (66.6%)	11 (84.6%)	0.312

*wk* weeks

Categorical variables are presented as n (%), and continuous variables as mean ± standard deviation (SD). Cervical length was measured by transvaginal ultrasound. Delta cervical length represents the difference between post-cerclage and pre-cerclage cervical length. Progesterone refers to vaginal progesterone administration

Early preterm birth (< 34 weeks) occurred in 8/52 women (15.4%), with no significant difference between groups (*p* = 0.299). Overall, 84.6% of women (44/52) delivered at ≥ 34 weeks of gestation (ultrasound-indicated: 84.6%, 33/39; physical examination-indicated: 84.7%, 11/13).

**Fig. 1:**
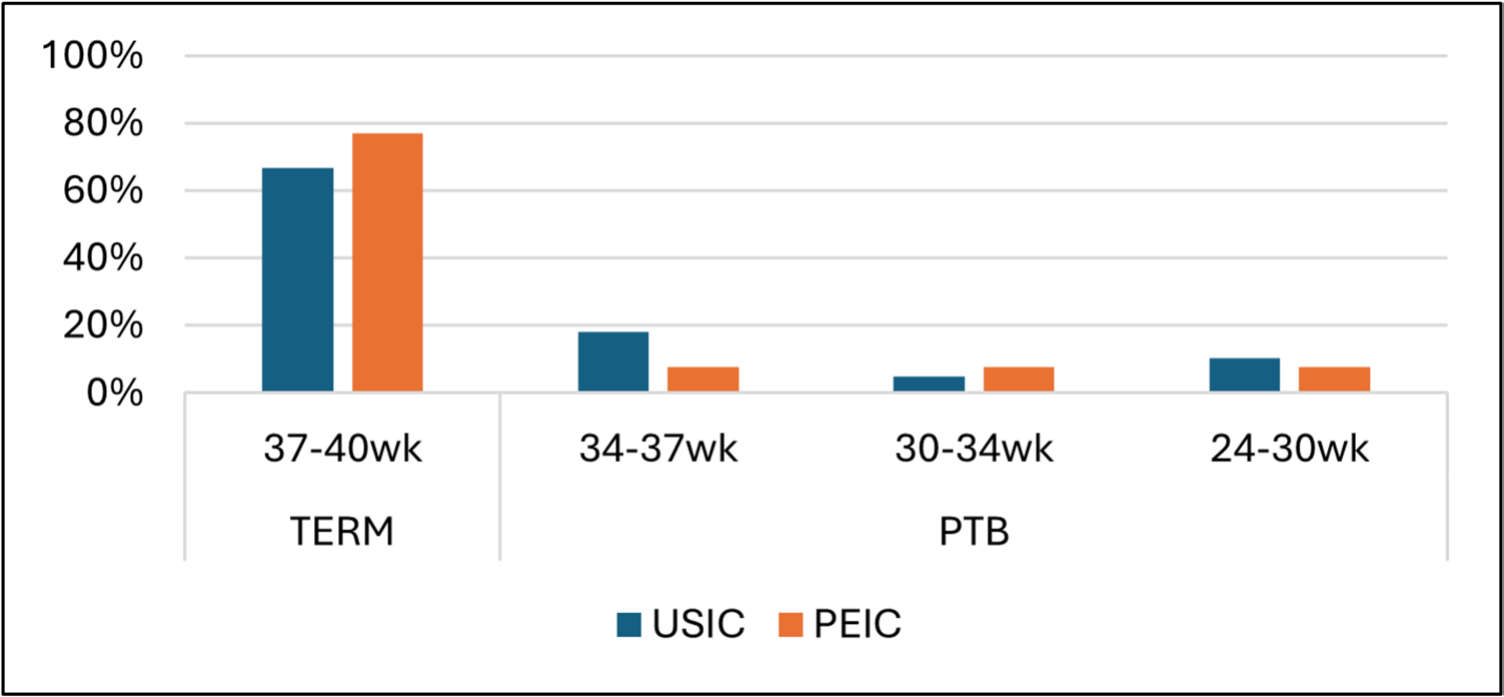
HYPERLINK "sps:id::fig1||locator::gr1||MediaObject::0" Gestational age at delivery after emergency cerclage

**Fig. 2:**
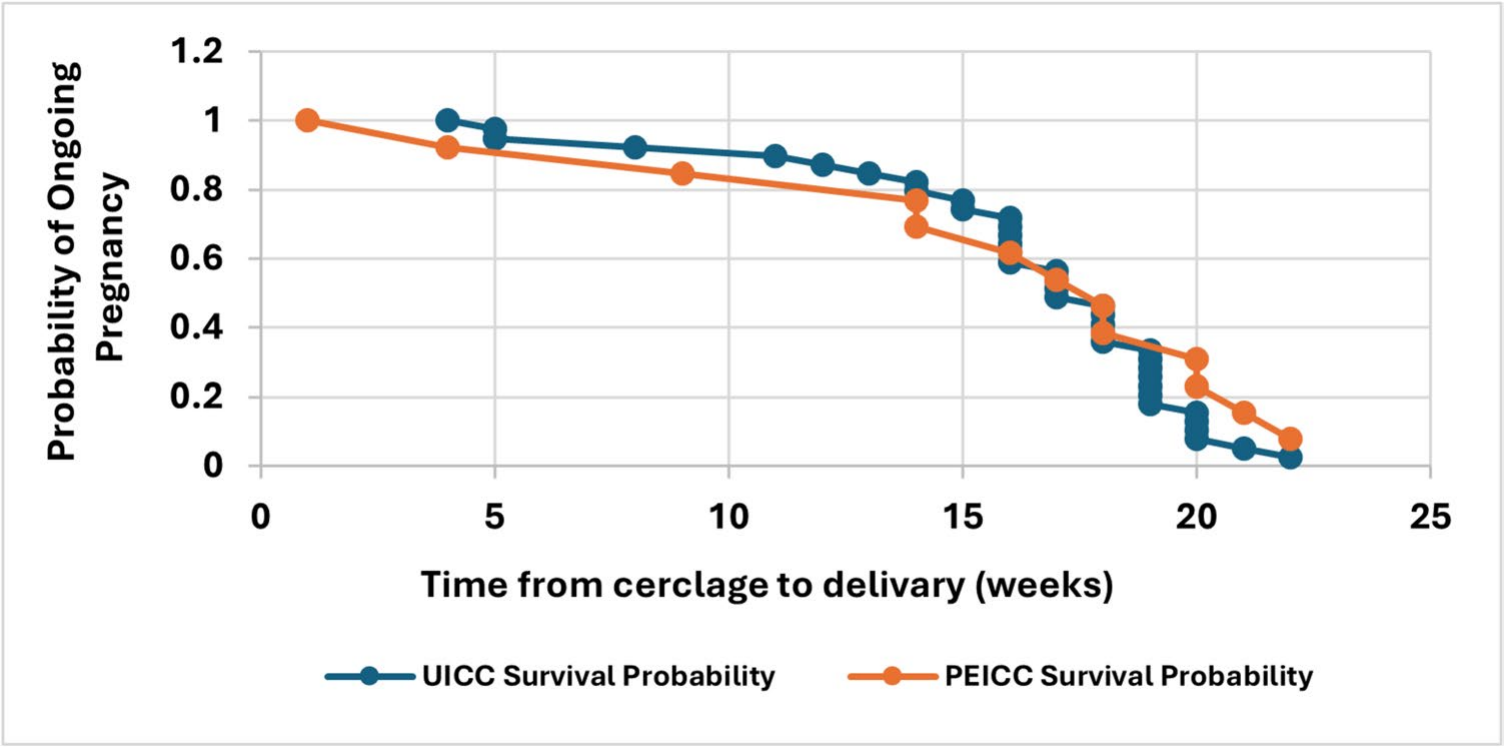
Kaplan–Meier curve showing time from cerclage to delivery according to cerclage indication

In multivariable logistic regression analysis adjusting for maternal age, parity, gestational age at cerclage placement, prior preterm birth, and history of hysteroscopy, cerclage indication was not independently associated with early preterm birth (aOR 0.45, 95% CI 0.04–5.20, *p* = 0.52). Pre-cerclage cervical length was independently associated with lower odds of early preterm birth (aOR 0.73 per 1-mm increase, 95% CI 0.56–0.96, *p* = 0.02). Gestational age at delivery by cerclage indication is presented in Fig. 1. In both groups, the majority of womendelivered at term (37–40 weeks), while preterm birth rates were relatively low. No statistically signifi cant diff erenceswere observed between groups.The time-to-delivery interval following cerclage placement is illustrated in Fig. 2. The Kaplan–Meier curves showsimilar probabilities of ongoing pregnancy over time in both groups, with no statistically signifi cant diff erenceobserved between them

## Discussion

In this study, emergency cervical cerclage, whether indicated by cervical shortening on ultrasound or cervical dilation on physical examination, was associated with substantial pregnancy prolongation and relatively low rates of early preterm birth. No statistically significant differences in pregnancy outcomes were observed between ultrasound-indicated and physical examination-indicated cerclage. These findings support, but do not confirm, the hypothesis that these presentations may represent stages along a pathophysiologic continuum of cervical insufficiency. However, the study was underpowered to detect moderate between-group differences; therefore, these findings should be interpreted with caution. Notably, in multivariable analysis, pre-cerclage cervical length, rather than indication category, was the only independent predictor of early preterm birth.

The terminology and assessment prior to cerclage placement are inconsistent in the literature; some studies rely primarily on ultrasound findings, whereas others incorporate physical examination [18, 23, 24]. We suggest that a standardized physical examination in all women considered for cerclage is essential to accurately define the indication and assess membrane status, thereby improving comparability across studies. The comparison between physical examination-indicated cerclage and ultrasound-indicated cerclage in terms of pregnancy outcomes has revealed significant differences in the literature [18, 24]. Physical examination-indicated cerclage is generally associated with poorer outcomes, including higher rates of preterm delivery, lower birth weights, and increased neonatal mortality [18, 24]. However, consistent with the results of our study, others have reported no statistically significant differences under selected conditions [25–27].

We recognize that patients with a short cervix without dilation and those with cervical dilation represent different baseline risk categories for preterm birth, with higher intrinsic risk in the dilation group. Nevertheless, we compared these groups to reflect the clinical spectrum of emergency cerclage and to assess whether standardized management may mitigate expected differences in outcomes. This approach reflects the clinical reality in which cervical shortening may precede painless cervical dilation, supporting the concept that these presentations may represent stages of the same disease process.

In the present study, emergency cerclage demonstrated a high success rate, with 84.6% of patients delivering beyond 34 weeks and a mean prolongation of approximately 15 weeks. This relatively favorable outcome may reflect selection bias, as patients with advanced infection or significant cervical dilation were excluded, and all procedures were performed by experienced clinicians.

Compared with prior reports [15, 26], our findings fall within the higher range of reported success rates, further supporting the potential benefit of timely intervention in carefully selected patients. From a clinical perspective, these findings underscore the importance of early recognition of cervical shortening and intervention prior to progression to advanced dilation, which may contribute to improved pregnancy outcomes. Previous studies have reported heterogeneous findings, with some demonstrating comparable outcomes between ultrasound- and physical examination-indicated cerclage when performed early and with appropriate patient selection [25, 27], whereas others reported poorer outcomes in the physical examination group (mean prolongation: 90 vs 37 days, respectively) [18, 24]. These inconsistencies with our study likely reflect differences in gestational age at cerclage placement, degree of cervical dilation (including cases with dilation > 4 cm), and inflammatory status at presentation. Our approach, which emphasized early intervention, exclusion of advanced infection, avoidance of cerclage in cases of significant cervical dilation, and consistent placement by experienced specialists, is in line with professional guidelines’ recommendations [23] and may explain the more favorable outcomes observed.

In our study, the physical examination-indicated cerclage group had a significantly shorter cervical length at diagnosis than the ultrasound-indicated group, reflecting more advanced disease. These findings support the concept that cervical length is a key marker of baseline risk. In multivariable logistic regression analysis, pre-cerclage cervical length remained independently associated with early preterm birth, whereas cerclage indication was not. This further supports the notion that disease severity, rather than the clinical classification itself, is the primary determinant of outcome. However, the wide confidence interval for cerclage indication suggests that the study may be underpowered to detect moderate between-group differences. Similarly, previous studies [28] have shown that outcomes following cerclage are strongly influenced by baseline disease severity, with physical examination-indicated cases reflecting more advanced cervical insufficiency rather than a distinct clinical entity. Consistent with this, pre-cerclage cervical length was the only independent predictor of early preterm birth in our multivariable analysis, suggesting that the degree of cervical remodeling may be more clinically relevant than the indication category itself. Residual confounding by indication may therefore persist despite adjustment. Stratified or sensitivity analyses based on cervical length were not feasible due to the limited sample size and would likely be underpowered.

These findings are consistent with previous reports and further support the role of cervical length and dilation as key markers of disease severity and predictors of preterm birth [26, 27, 29].

In our study, a significantly higher prevalence of prior hysteroscopy was observed in the physical examination-indicated cerclage group compared with the ultrasound-indicated group (46.2% vs. 25.6%, *p* = 0.035), suggesting a possible association between prior intrauterine procedures and more advanced cervical changes at presentation. However, our dataset did not allow us to differentiate between diagnostic and operative hysteroscopy, which may have different implications for cervical integrity. Although a direct causal relationship has not been definitively established, prior studies suggest that operative hysteroscopic procedures, particularly when repeated, may increase the risk of cervical insufficiency and preterm birth [30–32]. These findings indicate that repeated or complex operative hysteroscopies may adversely affect cervical competence, whereas uncomplicated diagnostic procedures likely carry minimal risk. However, this association remains uncertain and requires confirmation in larger prospective studies.

## Strengths and limitations

This study has several strengths, including a uniform surgical technique performed in a single tertiary center and standardized pre-procedural evaluation. However, important limitations must be acknowledged. First, the relatively small sample size, particularly in the physical examination-indicated group, limits statistical power and increases the risk of type II error. Second, the retrospective design introduces potential selection and information bias. Third, the comparison groups represent different baseline disease severity, raising the possibility of confounding by indication. Finally, the universal use of adjunct vaginal progesterone may have attenuated differences between groups, limiting the ability to isolate the independent effect of cerclage on pregnancy outcomes**.** Notably, a significant portion of the cohort was primigravida, which may have influenced the outcomes. Future studies involving larger and more diverse populations, with standardized protocols and prospective designs, are needed to confirm these findings and further evaluate the impact of indication type on pregnancy outcomes.

Additionally, the mode of delivery in our dataset was limited to the current pregnancy, and data on prior cesarean deliveries, including stage of labor, were unavailable; therefore, the potential contribution of second-stage cesarean delivery could not be assessed.

## Conclusion

Emergency cervical cerclage was associated with substantial pregnancy prolongation in this cohort of high-risk patients. No statistically significant differences in pregnancy outcomes were observed between ultrasound- and physical examination-indicated cerclage, and pre-cerclage cervical length, rather than indication category, was the only independent predictor of early preterm birth.

These findings support, but do not confirm, that ultrasound-detected cervical shortening and cervical dilation may represent different stages of the same pathophysiologic continuum of cervical insufficiency. Standardized terminology and careful clinical assessment, including physical examination, may help optimize patient selection. Further large, prospective studies are warranted to refine treatment protocols and unify clinical practice.

## Data Availability

The datasets generated and/or analyzed during the current study are available from the corresponding author on reasonable request.

## Declarations

### Use of AI-assisted technologies in the writing process

During the preparation of this manuscript, the authors used ChatGPT to assist with English language editing. All content generated with the aid of this tool was subsequently reviewed and revised by the authors, who take full responsibility for the final content of the publication.
